# Ibuprofen-Induced Hypokalemia and Distal Renal Tubular Acidosis: A Patient's Perceptions of Over-the-Counter Medications and Their Adverse Effects

**DOI:** 10.1155/2013/875857

**Published:** 2013-07-25

**Authors:** Mark D. Salter

**Affiliations:** ^1^The University of Sydney, NSW 2006, Australia; ^2^Emergency Medicine Department, Nepean Hospital, P.O. Box 63, Penrith, NSW 2751, Australia

## Abstract

We highlight a case of distal renal tubular acidosis secondary to ibuprofen and codeine use. Of particular interest in this case are the patient's perception of over-the-counter (OTC) medication use, her own OTC use prior to admission, and her knowledge of adverse reactions or side effects of these medications prior to taking them.

## 1. Introduction

Ibuprofen is a nonsteroidal anti-inflammatory drug (NSAID) which is available over-the-counter (OTC) as a nonprescription drug. It is used widely as an antipyretic and analgesic. Our patient had hypokalemia secondary to type one, distal renal tubular acidosis (dRTA), after long-term ibuprofen and codeine use. The combination of biochemical abnormalities including hypokalemia, hyperchloremic metabolic acidosis, hypophosphatemia, and urine pH >5.5 was consistent with those found in our patient [1–3]. 

The mechanism behind ibuprofen-induced renal tubular acidosis is not well understood and is believed to involve the inhibition or deficiency of carbonic anhydrase activity, especially carbonic anhydrase type two, which is the predominant form in the kidneys. Essentially, the hyperchloremic metabolic acidosis is a result of impairment of renal acidification and the hypokalaemia is due to the acidosis impairing proximal sodium reabsorption, causing increased potassium secretion in the distal tubules [4–6]. 

## 2. Case Presentation

A 38-year-old patient presents with myalgia, evolving paralysis, and vomiting for 3 weeks on a background of iron deficiency anaemia, migraines, and gastroesophageal reflux disease. Her only medications were esomeprazole 20 mg and amitriptyline 50 mg. She had been sent in by her GP with moderate hypokalaemia (potassium 2.6 mmol/L, range 3–5 mmol/L) and possible myositis (CK 2500 u/L) after blood tests the prior evening. Her examination demonstrated conjunctiva pallor, minor right upper quadrant, and epigastric tenderness. Neurological exam showed generalised upper and lower limbs flaccid weakness (grade 3/5 proximally and 4/5 distally) with normal sensation and cranial nerve examination. Repeat blood tests demonstrated a hyperchloremic metabolic acidosis, hypokalemia (potassium 2.1 mmol/L), a mild transaminitis, an inflammatory response (WCC 27.1 × 10^9^/L and procalcitonin 108 ug/L with normal CRP), and a creatinine kinase 26,100 u/L. This was in the context of normal renal function (urea 5.4 mmol/L and creatinine 85 umol/L). Urinary myoglobin was 266,900 ug/L, pH 9, and spot urinary potassium 22 mmol/L (overall >20 mmol over a 24-hour period). ECG showed mild ST depression without U waves and a CT-Abdomen was essentially normal. All biochemistry was consistent with type 1 distal renal tubular acidosis (dRTA) with rhabdomyolysis (Table 1) and a possible differential of undifferentiated myositis. She was reviewed by an emergency registrar, two emergency consultants, a surgical registrar, a medical registrar and an intensive care registrar and denied any nonsteroidal anti-inflammatory or laxative use when asked specifically about these medications with these exact words. She was taken to ICU and had both oral and intravenous potassium replacement totalling 430 mmol over the next 36 hours with intermittent use of sodium bicarbonate to maintain urine alkalinisation. Remarkably, 8 hours after ICU admission she complained of a previously undisclosed dental problem and admitted to a second intensive care registrar that she was taking 1.2–2 g of nurofen (ibuprofen) and 8 fiorinal dental capsules (paracetamol 500 mg, codeine phosphate 10 mg, and doxylamine succinate 2 mg) daily for the past five weeks. This combination of ibuprofen and codeine is likely the cause of the dRTA. With cessation of ibuprofen and codeine, adequate replacement, and other supportive therapy, our patient was discharged from ICU after 48 hours.

## 3. Discussion

What made this case stand out for us was not the significant medical process occurring for the patient but the perceptions of our patient in regard to her ibuprofen and codeine use. We realised after exhaustive review that because she did not understand them to be relevant to her ongoing condition and that she did not consider these tablets “medications” because they were OTC and easily accessible. She unknowingly missed the link between their use and her subsequent decline in health, and by not reporting their use, her ongoing care was compromised.

The importance of patient's perceptions surrounding nonprescription medications and what constitutes a medication is poorly understood within the medical community. As physicians we assume that our patients' understanding of the medications they are taking would prevent them from being used inappropriately and that they recognise that nonprescription medications may also have detrimental effects. There is ample evidence in the literature to show how misuse or prolonged use of these medications can cause substantial morbidity and mortality, with paracetamol and NSAIDs among those being regularly reported to cause liver failure, hypokalaemia, and gastrointestinal bleeding and perforation [7–9]. 

The literature on this subject also points towards the general public's lack of understanding of ibuprofen adverse effects, and other OTC medications adverse effects, as a whole. This includes the potential harms and serious side effects of these medication types which may occur without the patients linking the cause and effect. Wilcox et al. in two collated surveys of patients taking OTC and prescription NSAIDs showed that ibuprofen was the most frequent NSAID analgesic used due to being an OTC medication. Over 60% were unaware of the potential adverse effects of this drug and more than a quarter used more than the recommended daily dose [10]. This finding was strengthened in a survey of 183 patients by Ngo et al. which found that 65% of people did not seek medical advice prior to ibuprofen use, 66% had never read the manufacturer's printed warning instructions on potential interactions or adverse reactions, and 71% had used it for over 1 year [11]. Of note, a study in Northern Ireland in regard to patients' perceptions of OTC medications, including the misuse or abuse of these preparations found that over 86% would always follow the product directions and were very aware of the potential for abuse and adverse effects of the OTC medications they were using. This finding is in direct contrast to most of the published literature and is likely an outlier [12]. It has also been shown that in general patients had poor knowledge of the potential side effects of their medication but could still identify these problems as they occurred, unlike our patient who did not make the connection between her use and the hypokalaemia-induced dRTA [13]. 

Interestingly, our patient did not directly report the use of nurofen or fiorinal dental capsules as her belief was that their use was as a short-term analgesic for a preexisting dental problem. She did not anticipate their harmful effects over a prolonged period and finally reported their use as an incidental disclosure after suffering from her preexisting but unreported dental pain in the intensive care unit. Of significant importance in this case was the fact that she had multiple patient-doctor interactions, was asked about regular medication use on multiple occasions, and, despite this, was only asked for generic use of ibuprofen rather than brand names, like nurofen. This highlighted the importance of specific questioning if a class of medication is suspected to be causing the clinical illness. It has been shown that patients are willing and generally happy to disclose their OTC medication use if asked and will readily try OTC medications on physician advice [14]. These findings and many patients' lack of understanding of potential adverse effects of the OTC medications they use have previously been demonstrated in paediatric settings, especially regarding cough and cold syrups [15]. 

This case illustrated the need for physicians to be more suspicious of OTC medication use and the possible adverse reactions associated with them. It also shows that we as physicians should not be afraid to enquire about their use and to educate our patients on the adverse effects of these drugs when used correctly or abused. Taylor et al. highlighted in their small study that if the framing of discussion into the risk of adverse effects of OTC medications was done in a positive way, then this increased the likelihood of the patient taking the medication and adhering to the directions on the label, despite previous adverse effects with the same or similar drugs [16]. 

## 4. Conclusion

This case highlighted a type1 dRTA secondary to ibuprofen and codeine use and demonstrated that patient perceptions of OTC medications and their adverse reaction profile dictated the level of clinical suspicion of the treating physicians. Multiple studies in the literature demonstrate that patients may not perceive these medications as drugs that treating physicians should be aware of at the time of review and likely do not fully comprehend the adverse reactions when taking them. We have shown that education about OTC medications and their adverse effects is a positive development in patient care and that suspicion of use must be followed with specific rather than broad interrogation. We have further shown that in an age of multiple patient-doctor interactions we must always take care to illicit information at the level of the patient we are reviewing.

## Figures and Tables

**Table 1:** Biochemistry results on presentation.

	Numbers
Venous blood gas	
pH	7.25
pO_2_ (mmHg)	29
pCO_2_ (mmHg)	33
HCO_3_ (mmol/L)	14
Base excess (mmol/L)	−12
	
Full blood count	
Hb (g/L)	109
WCC (×10^9^/L)	27.1
Platelets	573
Haematocrit	0.36
Procalcitonin (ug/L)	108
CRP (mg/L)	5
ESR (mm/hr)	9
Electrolytes	
Na^+^ (mmol/L)	141
K^+^ (mmol/L)	2.1
Cl^−^ (mmol/L)	115
HCO_3_ (mmol/L)	15
Urea (mmol/L)	5.4
Creatinine (umol/L)	85
Anion gap (mmol/L)	13
Thyroid function tests	
TSH (mIU/L)	1.51
T4 (pmol/L)	14
Liver function tests	
Bilirubin (umol/L)	9
Albumin (g/L)	39
AST (U/L)	413
ALT (U/L)	159
GGT (U/L)	27
ALP (U/L)	134
Calcium/magnesium/phosphate	
Ca^2+^ (mmol/L)	2.28
Mg^2+^ (mmol/L)	1.05
PO_4_^2−^ (mmol/L)	0.63
Creatinine kinase U/L	26,135
Urine tests	
Urine Na^+^ (mmol/L)	105
Urine K^+^ (mmol/L)	22
Urine pH	9
Urine protein (g/L)	0.61
Urine creatinine (mmol/L)	2
Urine osmolality (mmol/kg)	218
Urine myoglobin (ug/L)	266.900
Urine protein/creat. ratio (mg/mmol creat.)	299
